# MicroRNA Expression in Malignant Pleural Mesothelioma and Asbestosis: A Pilot Study

**DOI:** 10.1155/2017/9645940

**Published:** 2017-07-03

**Authors:** Paola Mozzoni, Luca Ampollini, Matteo Goldoni, Rossella Alinovi, Marcello Tiseo, Letizia Gnetti, Paolo Carbognani, Michele Rusca, Antonio Mutti, Antonio Percesepe, Massimo Corradi

**Affiliations:** ^1^Molecular Genetics, Department of Medicine and Surgery, University of Parma, Parma, Italy; ^2^Thoracic Surgery, Department of Medicine and Surgery, University of Parma, Parma, Italy; ^3^Department of Medicine and Surgery, University of Parma, Parma, Italy; ^4^Medical Oncology, University Hospital of Parma, Parma, Italy; ^5^Pathological Anatomy and Histology, University Hospital of Parma, Parma, Italy

## Abstract

**Background:**

The identification of diagnostic/prognostic biomarkers for asbestos-related diseases is relevant for early diagnosis and patient survival and may contribute to understanding the molecular mechanisms underlying the disease development and progression.

**Aims:**

To identify a pattern of miRNAs as possible diagnostic biomarkers for patients with malignant pleural mesothelioma (MPM) and asbestosis (ASB) and as prognostic biomarkers for MPM patients.

**Methods:**

miRNA-16, miRNA-17, miRNA-126, and miRNA-486 were quantified in plasma and formalin-fixed paraffin-embedded samples to evaluate their diagnostic and prognostic roles compared to patients with other noncancerous pulmonary diseases (controls). Results. The expression of all the miRNAs was significantly lower in patients with MPM and ASB than that in controls. miRNA-16, miRNA-17, and miRNA-486 in plasma and tissue of MPM patients were significantly correlated. Furthermore, the expression of miRNA-16 in plasma and tissue, and miRNA-486 only in tissue, was positively related with cumulative survival in MPM patients.

**Conclusions:**

All the miRNA levels were decreased in patients with MPM or ASB, supporting the role of circulating miRNAs as a potential tool for diseases associated with exposure to asbestos fibers. miRNA-16 was directly related to MPM patient prognosis, suggesting its possible use as a prognostic marker in MPM patients.

## 1. Introduction

Long-term exposure to asbestos is the cause of some typical malignant and benign diseases, as malignant pleural mesothelioma (MPM) and asbestosis. MPM is a tumor originating from the mesothelial surfaces of the lung and is characterized by a poor prognosis. Chronic inflammation and genetic predisposition are concurrent factors in MPM pathogenesis. The silent clinical progression leads to a very late diagnosis, which strongly limits the therapeutic intervention and the extreme resistance to current chemotherapeutic agents. The diagnosis is histological and/or radiological and allows a median survival time of 9-10 months [1]. Asbestosis is a chronic lung disease caused by the inhalation of asbestos fibers. It is characterized by inflammatory response and production of free radicals, with consequent cytotoxic effects and stimulation of the proliferation and activation of fibroblasts in the interstitium. The deposition of collagen in the interstitium promotes the thickening of the bronchial and alveolar wall and, in short, diffuses interstitial fibrosis [2].

The identification of diagnostic biomarkers for MPM and asbestosis is relevant for early diagnosis and patient stratification and may give a contribution to understanding the molecular mechanisms underlying the development and progression of the tumor [3]. Many studies have shown that microRNAs (miRNAs) play an important role in regulating the development of several diseases in various organs, including the lung [4]. miRNAs are approximately 18–22 nucleotide RNAs that can recognize specific mRNA targets and regulate gene expression. They influence the transcriptional and posttranscriptional mRNA levels by promoting the degradation of their targets and/or suppressing translation [5].

The biogenesis of miRNAs is a multistep process that begins in the nucleus, culminates in the cytoplasm, and involves numerous enzymes and accessory proteins. miRNAs regulate various biological processes, such as cell differentiation, proliferation, metabolism, and apoptosis. A number of miRNA genes are located near sites of translocation breakpoints or deletions in various cancers. Therefore, miRNAs can act as tumor suppressors or oncogenes. A dysregulated miRNA expression has been observed in several diseases, including cancer [6–10].

In this study, we evaluated the expression of miRNA-16, miRNA-126, miRNA-486, and miRNA-17 in plasma and tissue samples of subjects with diagnosis of MPM or asbestosis with the aim to test them as possible biomarkers for the diagnosis of these two diseases and prognosis of MPM. A group of patients with benign pulmonary diseases was also included as negative controls. The choice of these miRNAs was based on two considerations: (1) their relevance in controlling important molecular pathways that may be implicated in MPM and (2) their relevance as biomarkers in other cancers.

Specifically, miRNA-16 is usually implicated in cancer development. miRNA-16 was firstly discovered in chronic granulocytic leukemia, and considered as a tumor suppressor gene [11, 12]. Its expression is strongly dysregulated in a variety of solid tumors such as breast, lung, and gastric cancer [13–15]. Furthermore, several articles have demonstrated the role of miRNA-16 in the control of cell cycle [16, 17].

miR-126 is related to the progression of several cancer types. miRNA-126 alters a number of cellular functions by suppressing translation of different target genes. It alters lung cancer cell phenotype by inhibiting adhesion, migration, and invasion [18, 19]. Furthermore, it inhibits cell proliferation, migration, and invasion in thyroid cancer cells [20], in osteosarcoma cells [21], in squamous cell carcinoma [22], and in colorectal cancer [23]. Finally, it is involved in the control of angiogenesis, an important molecular pathway implicated in the progression and metastasis of cancer [24–26].

miRNA-486 is downregulated in non-small-cell lung cancer (NSCLC) [27]. In addition, decreased expression of miR-486 was observed in tumor tissues from patients with lung, colon, melanoma, and gastric cancer [28–31]. Finally, the choice to evaluate miRNA-17 was an attempt to include a miRNA specifically related to MPM. The expression and function of miRNA-17 vary by cell type. It has become widely accepted that miRNA-17 has the potential to act either as an oncogene or as a tumor suppressor, depending on the cellular context [32–34]. Relative to MPM, both upregulation and downregulation were observed [35–37]. Importantly, its altered expression can involve a resistance to cancer therapy [38, 39].

## 2. Materials and Methods

### 2.1. Subjects and Clinical Specimens

The study involved 32 subjects with a diagnosis of MPM based on traditional diagnostic techniques (e.g., chest CT or X-ray) awaiting surgery, 14 subjects with a diagnosis of asbestosis, and 15 subjects with other noncancerous pulmonary diseases. In addition, 24/32 formalin-fixed, paraffin-embedded tissues (FFPE) of the subjects with MPM were used to correlate the biomarkers evaluated in the study. The patients' characteristics are summarized in Table 1. Considering the three study groups, there was no difference in terms of age, gender, or smoking habits (nonsmokers versus ex-/current smokers separately or together). Therefore, these variables were not included in further analyses. A venous blood sample was obtained from all participants at the time of diagnosis before receiving any treatment. Each patient gave his/her informed consent, and the study was conducted in conformity with the Declaration of Helsinki and approved by our local Ethics Committee.

### 2.2. Blood and Tissue Collection

Three milliliters of peripheral blood were collected in EDTA containing tubes (Becton, Dickinson and Company NJ, USA), processed within two hours, and centrifuged at 3000 rpm for 20 min. The plasma was then removed, and the samples were centrifuged again at 10000 rpm for five minutes in order to remove any cellular contamination. To exclude hemolysis in plasma, the samples were thoroughly inspected visually. The biopsy samples were routinely fixed in 10% neutral-buffered formalin, paraffin-embedded (FFPE) and dehydrated according to the standard protocols. The resulting FFPE and tissue blocks were stored at room temperature.

### 2.3. miRNA Isolation

RNA was extracted from 500 *μ*L of the plasma samples using the mirVana PARIS Isolation Kit (Life Technologies, Carlsbad, CA), in accordance with the manufacturer's instructions.

RNA was extracted from two 10 *μ*m slices of formalin-fixed paraffin-embedded tissues (FFPE) specimens using a Recoverall™ Total Nucleic Acid Isolation kit for FFPE (Life Technologies, Carlsbad, CA) in accordance with the manufacturer's instructions (Life Technologies, Carlsbad, CA).

The extracted RNA was digested with DNase I (DNA-free kit; Life Technologies, Carlsbad, CA) to remove any genomic DNA contamination. Total RNA was quantified using a NanoDrop spectrophotometer (Thermo Scientific, DE). Two independent RNA extractions were always performed to assess technical reproducibility.

### 2.4. Reverse Transcription and Quantification of miRNA Expression by q-PCR

The RNA was reverse transcribed using a TaqMan MicroRNA RT kit (Life Technologies, Carlsbad, CA) according to the manufacturer's instruction. The reaction included 3 *μ*L of stem-loop RT primer 50 nM, 1.5 *μ*L of 10x RT buffer, 0.15 *μ*L of dNTPs 100 mM, 0.19 *μ*L RNase inhibitor 20 U/*μ*L, 1 *μ*L of MultiScribe reverse transcriptase 50 U/*μ*L, and 5 *μ*L of RNA sample in a total volume of 15 *μ*L.

1.33 *μ*L plasma cDNA solutions were amplified using TaqMan 2x Universal PCR Master Mix (Life Technologies, Carlsbad, CA), a specific primer set, and hydrolysis probe-based Taq-Man microRNA Assays (Life Technologies, Carlsbad, CA) in 20 *μ*L of mixture. Quantitative PCR was performed using an iCycler iQ Real-Time Detection System (Bio-Rad, Hercules, CA). The reactions consisted of one step at 95°C for 10 min, followed by 40 cycles of 95°C for 15 s and 60°C for 1 min. All of the assays were made in duplicate, and one no-template and two interpolate controls were used in each experiment. The expression of the miRNAs was calculated using the comparative cycle quantitative (Cq) method. The quantitative cycle (Cq) was defined as the fractional cycle number at which the fluorescence passed the fixed threshold. The Cq values of the target miRNAs were normalized to Cq average of sno-RNU6B, sno-RNU44, and sno-RNU48 for tissue samples and to Cq of miRNA-146 for plasma samples. Small, noncoding RNAs, such as sno-RNAs and sn-RNAs, are typically used for data normalization in miRNA profiling experiments, in the same way that housekeeping genes are used for normalization of mRNA expression data [40]. We verified with specific experiments their validity as normalizing factors, confirming the literature (data not shown). However, sno-RNAs are not expressed in plasma samples. Since no consensus exists regarding the normalization in the serum/plasma to a standard reference miRNA in various diseases or experimental condition [41], we validated in this study the use of miRNA-146 as potential normalizing factor.

### 2.5. VEGF Plasma Quantification

Vascular endothelial growth factor (VEGF) was quantified in plasma samples using a commercially available competitive enzyme-linked immunosorbent assay (ELISA) in accordance with the manufacturer's instructions. The VEGF Quantikine Assay (Quantikine® R&D Systems, MN, USA) provides accurate measurements within the range of 31.2–2000 pg/mL and has a detection limit of 9 pg/mL. The samples were prepared in accordance with the extraction protocol suggested by the manufacturer.

### 2.6. Statistical Analysis

Sample size calculation started from two hypotheses: (1) a decrease of at least 50% in miRNA levels in at least 3 miRNAs in MPM versus controls and (2) a coefficient of variation of miRNA in controls of about 50% to take into account the nonnormality of data. Therefore, in a two-sided, two-sample *t*-test, a group sample size of 15 subjects per group was necessary to ensure an alpha value of 0.05/3 and a beta value of 0.20/3 (Bonferroni's correction).

IBM SPSS 21.0 statistical software (IBM, New York, NY) was used for the statistical analysis and graphs were generated using GraphPad Prism 4.0 (GraphPad Software Inc., La Jolla, CA). As the miRNA expression variables were not normally distributed (Kolmogorov-Smirnov test), Kruskal-Wallis's test followed by Dunn's post hoc comparison was used to compare the data depending on the number of tested groups. Median and interquartile range were therefore reported.

All the relationships between pairs of continuous variables were tested by means of simple linear regression models. The logarithm of miRNAs was always used to reduce the effects of outliers and normalize the residuals. The diagnostic power of miRNAs was tested by means of ROC curves. Area under the curve (AUC) was calculated with its 95% CI interval (a lower extreme >0.5 indicated a significant diagnostic power), while the cut-off point as the point which maximized the sum of sensitivity and specificity.

The relationship between miRNAs and cumulative survival was tested by using univariate Cox regression models, by using the logarithm of miRNAs as continuous factor. All of the tests were two sided and a significance level of *p* = 0.05 was fixed.

## 3. Results

**Figure 1:**
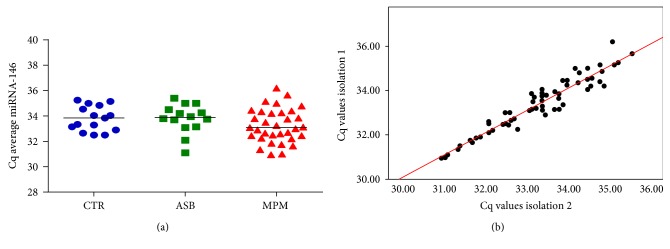
(a) Average Cq values for the miRNA-146 evaluated in the plasma samples of MPM, asbestosis patients, and the control group. No differences were found. (b) Reproducibility between technical replicates. Two independent RNA isolations and two qPCR were performed from the same plasma samples (*p* < 0.001; *R*^2^ = 0.911).

We firstly validated miRNA-146 as normalizing factor for plasma. As a first step, we evaluated the repeatability (intra-assay variation) of qPCR measurements. Each qPCR was performed three times, to evaluate the expression of the candidate reference gene in the patient and control group. The candidate reference miRNA displayed a minimal expression range. The mean quantification cycle (Cq) was 33.65 with a SD of 0.98 [%CV = 0.30]. Furthermore, the expression was unaltered in the three groups of patients (Figure 1(a)). Finally, the reproducibility between technical replicates was overall good (Figure 1(b)).

**Figure 2:**
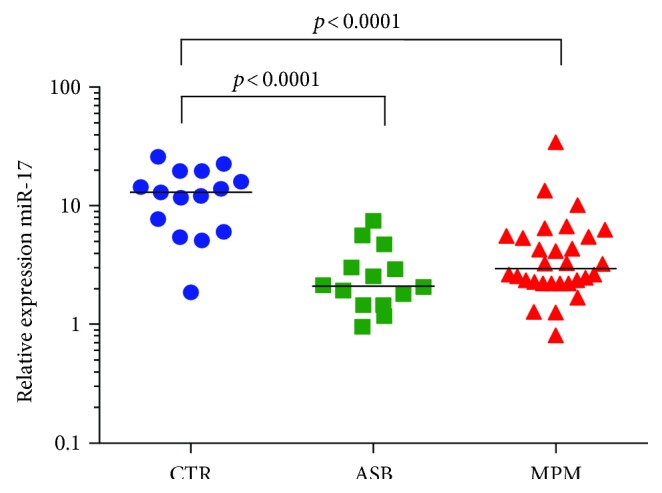
Plasma miRNA-17 quantification in controls (blue circle), ASB (green square), and MPM patients (red triangle). The median value is also reported.

**Figure 3:**
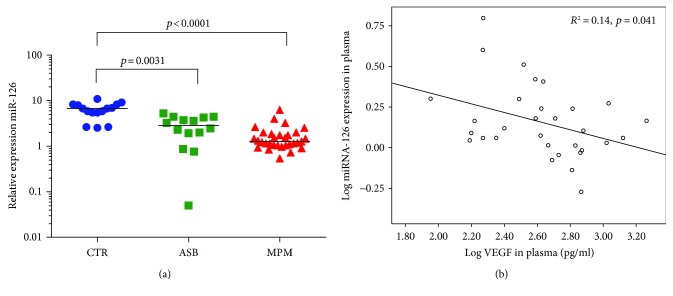
(a) Plasma miRNA-126 quantification in controls (blue circle), ASB (green square), and MPM patients (red triangle) with median values; (b) relationship between miRNA-126 and VEGF plasma.

**Figure 4:**
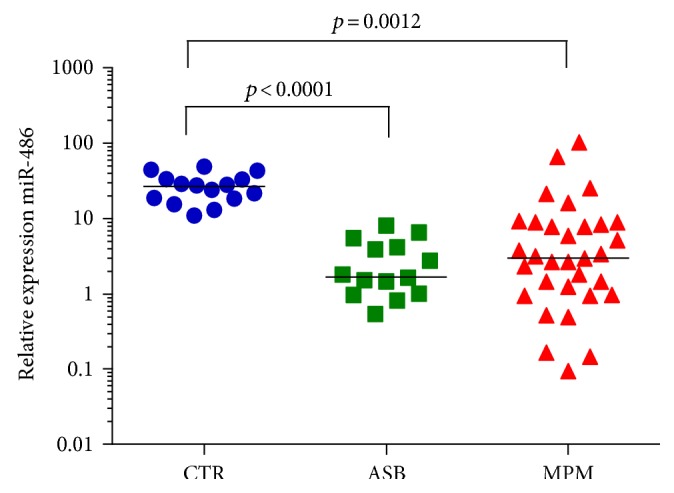
Plasma miRNA-486 quantification in controls (blue circle), ASB (green square), and MPM patients (red triangle), with median values.

**Figure 5:**
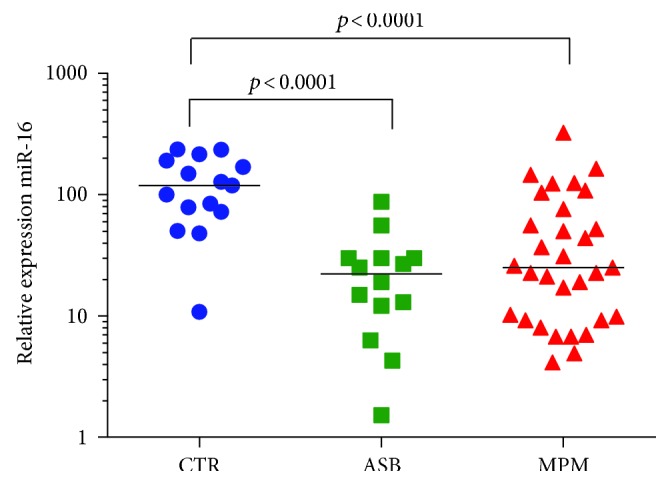
Plasma miRNA-16 quantification in controls (blue circle), ASB (green square), and MPM patients (red triangle), with median values.

miRNA-17 was significantly downregulated in the plasma of subjects with MPM (*p* < 0.0001) and asbestosis (*p* < 0.0001), whereas no differences were detected between the ASB and MPM groups [2.9 (2.2–5.5) in MPM patients, 2.1 (1.5–3.5) in ASB patients, and 13.0 (6.1–19.7) in controls (Figure 2)]. Similarly, miRNA-126 was downregulated in patients with MPM (*p* = 0.0001) and in patients with ASB (*p* = 0.003), but the two groups were not statistically different [1.3 (1.0–1.9) in MPM patients, 2.9 (1.6–4.3) in ASB patients, and 6.7 (5.5–8.3) in controls (Figure 3(a))]. Moreover, by studying the correlation between the miRNA-126 expression and VEGF concentration, a significant but weak inverse correlation was seen only in the MPM group (*p* = 0.041, *R* = −0.37, Figure 3(b)). VEGF concentrations were not different among the groups (data not shown). miRNA-486 had a similar trend of the previous miRNAs. Its plasma expression was significantly downregulated both in the MPM group (*p* = 0.012) and in the ASB group (*p* < 0.0001), but the two groups were not statistically different [2.9 (1.0–8.4) in MPM patients, 1.7 (1.0–4.3) in ASB patients, and 14.9 (9.5–22.6) in controls (Figure 4)]. Finally, microRNA-16 levels resulted significantly lower in the MPM group (*p* < 0.0001) and in the asbestosis group (*p* < 0.0001). No differences were detected between the ASB and MPM groups [25.1 (9.2–76.1) in MPM patients, 22.1 (10.7–29.9) in ASB patients, and 128.0 (84.5–190.7) in controls (Figure 5)].

Given the results, ROC curves were used to distinguish MPM + ASB patients (two overlapping groups) from controls. As reported in Table 2, the diagnostic power was always good.

**Figure 6:**
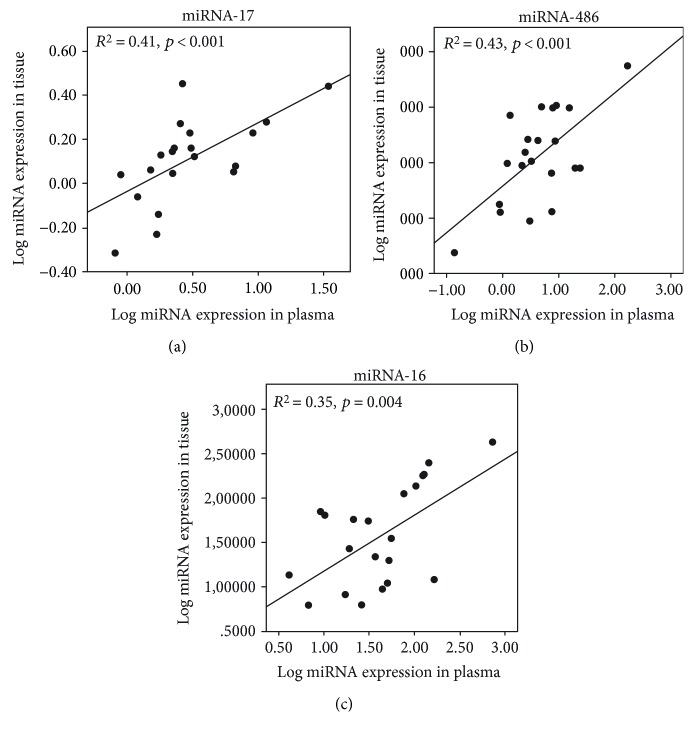
Correlation between tissue and plasma levels of (a) miRNA-17, (b) miRNA-486, and (c) miRNA-16 in patients with MPM.

miRNA-17 expression in plasma and tissue samples in the MPM group was correlated (*p* < 0.001, *r* = 0.64) (Figure 6(a)), as miRNA-486 (*p* < 0.001, *R* = 0.66) (Figure 6(b)) and miRNA-16 expression (*p* = 0.004, *R* = 0.59) (Figure 6(c)). No correlation was observed between plasma and tissue miRNA-126 expression.

Looking at the relationships between pairs of miRNAs in the same biological matrix, miRNA-16 and miRNA-486 in plasma and tissue were correlated (*r* = 0.75, *p* < 0.001 and *r* = 0.58, *p* = 0.004, resp., not shown).

The prognostic power of the miRNAs in plasma was tested by means of univariate Cox's regression model on MPM patients. Age, gender, and sex were not significant, and therefore, multivariate models were not performed at this stage. Among miRNAs, miRNA-16 in plasma (*p* = 0.05, HR = 0.35 [0.11–1.0] per change of 1 in the logarithm) and in tissue (*p* = 0.012, HR = 0.02 [0.001–0.42] per change of 1 in the logarithm) and miRNA-486 in tissue (*p* = 0.005, HR = 0.24 [0.09–0.64] per change of 1 in the logarithm) showed a significant relationship with cumulative survival. Finally, none of the evaluated microRNAs was related to the histopathological features of the tissues, although lower levels of miRNA-486 were highlighted in biphasic type with respect to epithelioid histological type, without however achieving statistical significance (data not showed).

## 4. Discussion

Malignant pleural mesothelioma is a fatal cancer, as current treatments rarely extend patient survival beyond 12 months [42]. The knowledge of the cytogenetic and molecular aspects of mesothelioma has progressed substantially in the recent years. Although the carcinogenic mechanisms of asbestos are not fully understood, during the latency period of several years, inhaled asbestos or other carcinogenic fibers end up in the pleura, inducing cytotoxicity, DNA damage, frustrated phagocytosis, and chronic inflammation [43]. Asbestos fibers are clastogenic and cytotoxic in vitro and induce abnormal segregation at mitosis. Their transforming activity, both in vitro and in vivo, has been related to fibers dimensions, durability, and surface properties [44]. Novel diagnostic biomarkers are currently being tried for clinical use, but there are no markers for early diagnosis, prognosis, or prediction of therapy.

Identifying tumor biomarkers that indicate the presence/absence of disease using noninvasive diagnostic procedures is a key part of cancer research. Recent data strongly support the idea that alterations in the expression of cell-free miRNAs circulating in body fluids are associated with the pathogenesis of various forms of cancer, including MPM [45–48].

Several reports have proposed the detection of circulating tumor-specific miRNAs as a potentially valuable tool for early cancer detection in order to predict the clinical cancer behavior and/or its therapeutic response.

In addition to mesothelioma, exposure to asbestos fibers can cause asbestosis. Asbestosis is a chronic lung disease characterized by a scarring of lung tissues, which leads to long-term breathing complications. The disease worsens slowly over time. Both diseases have the same etiology but a different clinical evolution. The purpose of this study was to identify biomarkers of mesothelioma disease and pick out a possible molecular mechanism leading towards one or the other pathology.

In this study, all investigated miRNAs were downregulated in the plasma of subjects with mesothelioma and with asbestosis with respect to the healthy subjects.

The human miRNA-17/20 cluster's genomic location, chromosome 13q31, correlates with loss of heterozygosity in several different cancers. The expression and function of miRNA vary by cell type. It has become widely accepted that miRNA-17 has the potential to act either as an oncogene or as a tumor suppressor, depending on the cellular context [49–54]. In this study, miRNA-17 was downregulated in MPM than in asbestosis subjects with respect to the controls. In light of this, our data corroborates the hypothesis that in mesothelioma, miRNA-17 behaves like a tumor suppressor. It was found in idiopathic pulmonary fibrosis samples that miRNA-17 was reduced and the introduction of miR-17 in idiopathic pulmonary fibrosis (IPF) lung fibroblasts reduced fibrotic gene [55]. miR-17 has been implicated also in liver fibrosis [56]. Our data show that miRNA-17 is significantly reduced in subjects with asbestos-correlated pathologies, corroborating the hypothesis of its involvement in the control of the development of the fibrotic component.

miRNA-486 is located at chromosome 8p11, a region of frequent genomic loss in multiple cancers. Decreased expression of miRNA-486 was observed in tumor tissues from patients with esophageal squamous cell carcinoma, breast, and gastric cancer [57–59]. Additionally, the downregulation of miRNA-486 was reportedly responsible for both tumor progression and metastasis by relieving the inhibition of protumorigenesis in lung cancer [60]. Decreased miRNA-486 expression was also linked to the progression of pulmonary fibrosis in both humans and mice. Decreased expression of miRNA-486 was observed in both silica- and bleomycin- (BLM-) induced mouse models of lung fibrosis, and miRNA-486 expression was decreased in tissue samples from patients with silicosis and IPF. In the study of Ji et al. [61], the overexpression of miRNA-486-attenuated pulmonary fibrosis in mice and repressed TGF-*β*1-induced fibrogenesis in NIH/3T3 cells, thus demonstrating that miR-486 has a strong antifibrotic activity in lung tissues and may be a novel target in the treatment of pulmonary diseases. Our results are in agreement with these data. Asbestosis is a disease, in which the fibrotic tissue becomes the main component of the lung tissue. Consequently, the broad involvement in fibrotic signaling events justifies the fact that miR-486 is strongly downregulated in patients with asbestosis. Moreover, the development of mesothelioma also leads to the development of a fibrotic process, and to further support this thesis, among the subjects with MPM, miRNA-486 was prevalently downregulated in the biphasic type, where the fibrotic component is the most abundant with respect to epithelial type. Based on these results, it could be assumed that miRNA-486 is used either as a novel marker in the diagnosis of asbestosis or as a possible target in the treatment of MPM.

miRNA-126 is an endothelial-specific miRNA that is located within intron 7 of epidermal growth factor-like domain 7 (EGFL7) [62, 63]. Several reports have described an oncogenic role for miR-126, such as the inhibition of apoptosis in acute lymphoblastic leukemia [64] and the promotion of gastric carcinogenesis [65]. Many studies have highlighted the strong downregulation of miRNA-126 in several cancer types such as pancreatic [66], lung, and kidney ones [67, 68]. miRNA-126 is often referred to as a tumor suppressor [69]. Angiogenesis has a pivotal role in chronic inflammation, tumor progression, and metastasis [70, 71]. Expression of VEGF and its receptors correlates with the degree of vascularization of many experimental and clinical tumors as detected by in situ hybridization and immunohistochemistry, and both have been used as prognostic indicators of an increased metastatic risk. It was reported that VEGF is a target gene of miRNA-126 and downregulation of miRNA-126 increases VEGF activity in lung [72, 73] and breast cancer [74]. The VEGF mRNA is miRNA-126 target, and studies have argued for a role of miRNA-126 in regulating VEGF-mediated signal transduction [74–76]. In line with these results, this study shows not only a downregulation of miRNA-126 in plasma samples of subjects with MPM but also an inverse correlation with VEGF expression in the same subjects, corroborating the hypothesis of an important role in the vascularization cancer process.

MicroRNA-16 (miRNA-16) has been demonstrated to regulate proliferation and apoptosis in many types of cancer. miRNA-16 is located at chromosome 13q14, which is deleted in 68% of chronic lymphocytic leukemia cases and is downregulated in most solid tumors. Several studies found that miRNA-16 influenced the expression of numerous genes in human cell lines [77, 78].

Particularly, miRNA-16 reduced cell proliferation and migration and caused G0/G1 cell cycle arrest, thus behaving as a tumor suppressor. Recent data have shown that miRNA-16 regulates cyclin D1 and cyclin E expression through their 3'UTRs [79, 80]. miRNA-16 has emerged as an especially key miRNA in the G1 cell cycle checkpoint. miRNA-16 targets several cell cycle regulators, including cyclin D1 (CCND1), cyclin D2 (CCND2), cyclin D3 (CCND3), cyclin E1 (CCNE1), cyclin-dependent kinase-1 (CDK1), and cyclin-dependent kinase-6 (CDK6).

Linsley et al. found that miRNA-16 induced an accumulation of A549 cells in G0/G1, and a similar cell cycle phenotype in MCF7 (breast cancer) and TOV21G (ovarian cancer) cells. Among miRNA-16-downregulated targets are many known cell cycle regulators that did not induce G0/G1 cell accumulation but may modify the effects of other targets (i.e., E2F7, CDC25A, CHEK1, WEE1, and CCNE1 genes) [80]. Coordinate regulation of these and other cell cycle regulators suggest highly orchestrated cell cycle control by miRNA-16. Reid et al. [81] report that, compared with normal mesothelium or mesothelial cell lines, a consistent downregulation of the miR-15/16 family was observed in all MPM tumors and cell lines investigated. Restoring miR-16 in vitro resulted in growth inhibitory and drug sensitizing effects in MPM cell lines, but not normal mesothelial cells. In our opinion, these data are in accordance with our results, in which we have highlighted that MPM patients with higher expression level of miRNA-16 show a higher survival, probably due to a greater control over the cell cycle exercised by this miRNA and corroborating the hypothesis of an important role of this miRNA in the process of carcinogenesis. The role of miRNA-16 in the control of several genes implicating in the migration and proliferation of cancer cells justify the fact that upper values were in subjects with higher survival. In our opinion, if this result will be confirmed in larger studies, the miRNA-16 could be a useful clinical use as prognostic biomarkers in mesothelioma therapy. And considering that MPM is remarkably resistant to therapy, these results may have promising implications in the future.

## 5. Conclusions

Although the sample size used in the study was small, the noninvasive characteristics of plasma sampling lead us to hypothesize that these biomarkers could be used alongside traditional imaging techniques in clinical practice and may help in the early diagnosis of MPM. The available data clearly support the role of miRNAs in the etiology of mesothelioma and asbestosis suggesting their possible use as diagnostic/prognostic markers of disease. However, a large-scale, multicenter clinical project is required to validate their usefulness before they can be adopted in routine clinical settings and used as noninvasive confirmatory screening tests that are complementary to imaging procedures. Besides, considering that chemotherapy, together with surgery, is the only option, which allows to delay the inexorable progression of the disease, it is possible to hypothesize a use of these miRNAs as a new molecular target for therapy. Considering that miRNAs are the underlying mechanism in the development of human disease, regulation of miRNA function may have therapeutic utility. miRNA-based therapeutics is an attractive research area and is a promising field to improve the treatment of cancers like MPM and other diseases.

## Figures and Tables

**Table 1:** Clinical features of the patients under study.

	Controls (*n* = 15)	MPM (*n* = 32)	ASB (*n* = 14)
Gender	12 M/3 F	24 M/8 F	8 M/6 F
Age (years)	69.9 (SD: 7.0)	72.3 (SD: 15.6)	75.9 (SD: 9.9)
Smoking history	5 NS 7 S 3 EXS	12 NS 20 S	8 NS 4 S 2 EXS
Diagnosis	8 nodules 3 bronchiectasis 4 other†	26 epithelioid 6 biphasic	
Stage		2 I 9 II 15 III 6 IV	

NS: nonsmokers; EXS: ex-smokers; S: smokers; SD: standard deviation; †Inflammatory disease (n. 2), aspiration pneumonia (n. 1), and rhino-bronchial syndrome (n. 1).

**Table 2:** Main parameters of ROC curves of the miRNAs in plasma. ^∗∗^ = *p* < 0.001.

	AUC (95% CI)	Cut-off value	Sensibility specificity at cut-off
miRNA-17	0.88^∗∗^ (0.78–0.98)	5.9	80.0–84.4
miRNA-126	0.95^∗∗^ (0.89–1.00)	5.4	80.0–97.8
miRNA-486	0.88^∗∗^ (0.79–0.96)	9.2	80.0–89.1
miRNA-16	0.89^∗∗^ (0.81–0.97)	77.5	86.7–82.2
